# Patterns of Gut Bacterial Colonization in Three Primate Species

**DOI:** 10.1371/journal.pone.0124618

**Published:** 2015-05-13

**Authors:** Erin A. McKenney, Allen Rodrigo, Anne D. Yoder

**Affiliations:** 1 Department of Biology, Duke University, Durham, North Carolina, United States of America; 2 National Evolutionary Synthesis Center, Durham, North Carolina, United States of America; 3 Duke Lemur Center, Duke University, Durham, North Carolina, United States of America; Charité, Campus Benjamin Franklin, GERMANY

## Abstract

Host fitness is impacted by trillions of bacteria in the gastrointestinal tract that facilitate development and are inextricably tied to life history. During development, microbial colonization primes the gut metabolism and physiology, thereby setting the stage for adult nutrition and health. However, the ecological rules governing microbial succession are poorly understood. In this study, we examined the relationship between host lineage, captive diet, and life stage and gut microbiota characteristics in three primate species (infraorder, Lemuriformes). Fecal samples were collected from captive lemur mothers and their infants, from birth to weaning. Microbial DNA was extracted and the v4 region of 16S rDNA was sequenced on the Illumina platform using protocols from the Earth Microbiome Project. Here, we show that colonization proceeds along different successional trajectories in developing infants from species with differing dietary regimes and ecological profiles: frugivorous (fruit-eating) *Varecia variegata*, generalist *Lemur catta*, and folivorous (leaf-eating) *Propithecus coquereli*. Our analyses reveal community membership and succession patterns consistent with previous studies of human infants, suggesting that lemurs may serve as a useful model of microbial ecology in the primate gut. Each lemur species exhibits distinct species-specific bacterial diversity signatures correlating to life stages and life history traits, implying that gut microbial community assembly primes developing infants at species-specific rates for their respective adult feeding strategies.

## Introduction

Research on the human gut microbiome (GM) has burgeoned in the past decade [1–9]. These studies have revealed that the GM has profound impacts on phenotype, ranging from cognition [10] to locomotion [11]. Given that humans represent but a single species, however, these studies are unable to establish the general evolutionary and ecological "rules" by which the GM is developed and maintained. Despite intensifying scientific interest and enhanced technical feasibility, it remains difficult to tease apart the relative significance of host characteristics in shaping the GM and to determine how and to what extent microbial niches and succession are determined by phylogeny or ecology. Although it has been shown that dietary intake can radically alter the composition and efficacy of the GM [12], the mechanisms by which these shifts are produced remain uncharacterized. Here, we study the composition and community development of GMs across multiple species within a phylogenetically related but ecologically diverse group of mammals. Whereas other recent research on primate microbiomes has relied on limited cross-sectional sampling [3,6–8,12–17] we present an extensive longitudinal study of the GM diversity of mother-infant pairs of three different lemur species from birth to weaning.

Our focal host species are within the primate clade Lemuriformes, which (together with the lorisiform primates) is sister to the haplorrhine primates (monkeys, apes and humans). In our study system, species are fed captive dietary regimes formulated to approximate the nutritional composition of natural diets consumed by conspecifics in the wild. The macronutrient composition within each species’ captive diet remains constant, while food items may be substituted to accommodate individual food preference and seasonal availability. Lemurs have evolved diverse GIT morphologies to adapt to species-specific feeding ecologies, making them an ideal group for teasing apart the potentially divergent effects of phylogenetic history and captive diet on GM community structure and for examining the associated community signatures for potential effects on host health and nutritional uptake.

### Gut microbial ecology

The mammalian gastrointestinal tract (GIT) is characterized by extremely low diversity at birth, but by adulthood, it teems with trillions of microbes that perform a variety of functions including fiber digestion and defense against pathogens [8,12]. Colonization of the GIT is initiated during birth and thus affected by the mode of delivery [18]. Progressive succession within the human infant GIT corresponds with key stages of development such as nursing, weaning, and intestinal development and maturation [1,3,6–8,12–17], in addition to life events such as antibiotic or novel dietary regimes [7,8]. Given the crucial role the GM plays in infant development, its permanent impact on the neonatal immune system [19] and brain development [10], and the observed persistence of gut bacterial strains across years of sampling [4], colonization events have the power to impact the host for its entire lifecycle. It is therefore likely that GMs are inextricably tied to life history (growth and reproduction rates, reproductive duration; [20]).

Gut microbes themselves are governed to varying extents by several host characteristics. First, genetically derived differences in immuno-“tolerance,” affect the host’s ability to recognize microbes as either helpful or harmful to the host [21]. Second, the host’s nutritional intake contributes to available microbial niche space [10,15,19,22,23]. Third, gut morphology provides a more or less complex topography for the microbes to colonize. After maternal inoculations initiate the infant GM during birth, diversity and membership increase with age and the introduction of solid foods [8,15]. Opportunistic pioneer species alter the GIT environment and create additional niche space in the form of fermentation byproducts [24]. These metabolites can be used by other microbes and thus help to drive succession.

After the assembly process is complete, the stable GM climax community prevents further colonization (i.e. by pathogenic microbes), possibly due to the functional redundancy among bacterial species [25]. Once established, the healthy adult human climax GM remains fairly stable except to reflect changing environmental conditions or health [26]. Of particular interest, Faith et al. [4] detected fluctuations in human and other mammals’ GM membership and metagenomic profiles in response to diet. Researchers have also observed significant shifts in response to global relocation, illness, and antibiotics [26,27].

### Our study system

**Fig 1:**
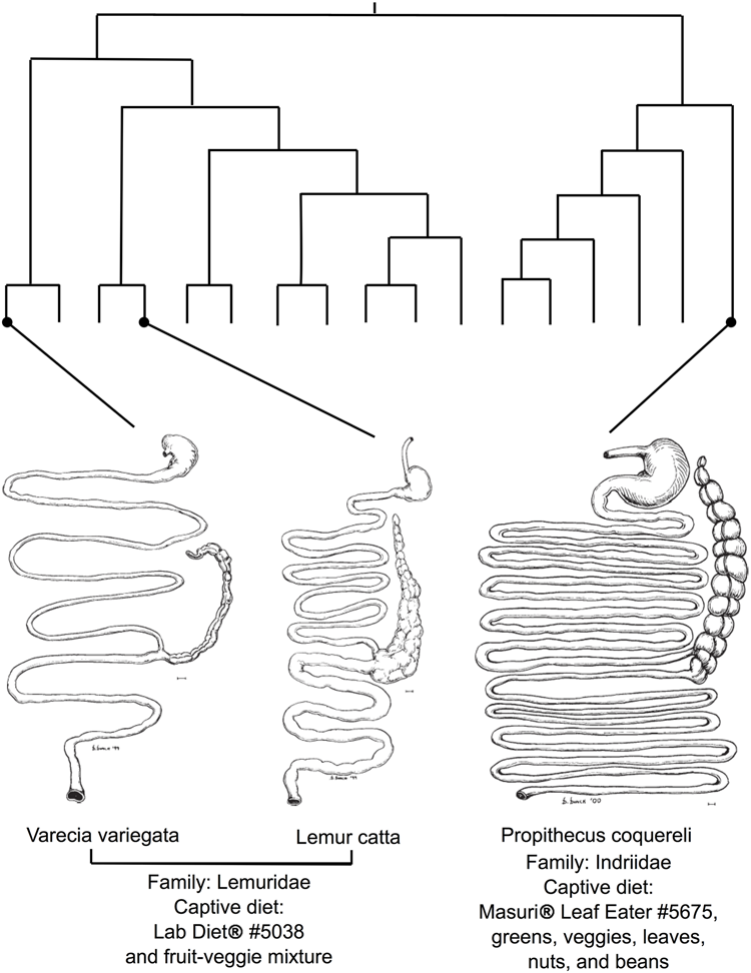
GIT diagrams [49] and feeding strategies for (A) *Varecia variegata*, *(B) Lemur catta*, and (C) *Propithecus sifaka*, projected onto a phylogenetic tree [28]. H: If the succession process in lemurs is similar to that described in humans, then microbial diversity should be lowest in samples from birth and increase with age until weaning, with decreasing intraspecific variability as individuals approach adulthood and their GMs approach the climax community. We refer to this as the “life stage” hypothesis.

We examine GM assembly in *Varecia variegata*, *Lemur catta*, and *Propithecus coquereli* (see Fig 1). Estimated divergence times are ~32mya for the divergence of *V*. *variegata* and *L*. *catta* and ~ 42mya for the divergence of *P*. *coquereli* from the Lemuridae (*V*. *variegata*, *L*. *catta*; [28]). Each species has evolved unique and differing life histories, ecological constraints, and distinct GIT morphologies. In our study system, however, *V*. *variegata* and *L*. *catta* are fed similar diets in captivity. We analyzed bacterial 16S rDNA reads amplified from fecal samples collected from positively identified individuals to resolve GM membership at the genus level and to compare succession patterns between individuals and across species. By controlling certain environmental variables such as dietary intake and recording relevant long-term metadata, we investigate the contributions of host life stage, captive diet, and GIT morphology to the host-gut microbial relationship and the process of community assembly. We use summary statistics of taxonomic richness, membership, and diversity to test the following hypotheses.

## Materials and Methods

### Animal housing and husbandry

All animals were housed at the Duke Lemur Center in Durham, NC, USA. Conspecific lemurs are housed in indoor enclosures with access to adjoining outdoor runs. This typical indoor cage is 10 feet high x 7.5 feet wide x 7 feet long (approximately 3 m high x 2.3 m wide x 2.1 m long). The typical outdoor enclosure is 10 feet high x 7.5 feet wide x 14 feet long (approximately 3 m high x 2.3 m wide x 4.3 m long). Each lemur social group is allocated enough cages that each individual could have his/her own indoor and outdoor area if needed. For example, a group of five animals would be able to move between 5 connected indoor and 5 connected outdoor enclosures.

Because at least half of the space is outdoors, and each indoor enclosure has 1–3 windows, lighting conditions follow the natural North Carolina photoperiod. Fluorescent lighting is also used from approximately 7am-5pm while staff are present. Social groups are provided a variety of enrichment items, including bamboo or wooden climbing structures, sturdy plastic play houses, elevated plastic nesting boxes or crates, firehoses and ropes for climbing, and rotating novel objects (i.e. scents, paper mache objects, puzzles with treats inside, etc.).

All infants are housed with their mothers, separate from other lemurs, immediately after birth and during nursing to reinforce the mother-infant bond. In this study, one (*L*. *catta*) social group contained two mother-infant groups (with three infants total). However, dams were never observed to nurse infants other than their own offspring, so this arrangement did not impact vertical transmission via milk.

### Captive diets

All individuals within each species are fed similar dietary regimes. *V*. *variegata* and *L*. *catta* are fed similar captive diets in alignment with their phylogenetic relatedness compared to *P*. *coquereli* (though in nature, their diets differ in the degree to which fruit is represented, with *V*. *variegata* being more frugivorous than *L*. *catta*). The following are representative dietary regimes per individual. *V*. *variegata* receive 80–100g Lab Diet #5038 plus approximately 145g fruit-veggie mixture. *L*. *catta* receive 60g Lab Die #5038 plus approximately 110g fruit-veggie mixture. *P*. *coquereli* receive 75g Mazuri Leaf Eater #5675, 30g greens, 30g veggies, 10g nuts/beans, and 150g leaves. Amounts may vary depending on individual health, weight, and reproductive status.

### Sample collection

All animal procedures were reviewed and approved by the Duke University IACUC under protocol number A282-11-11. Fifty-eight fresh fecal samples were collected from subjects at the Duke Lemur Center (Durham, NC, USA), either during routine handling or from enclosures immediately after excretion. Samples were collected from a total of 6 dams within one day of parturition and from a total of 9 infants at the following life stages: birth, nursing, introduction of solid food, routine ingestion of solid food, weaning, and three months post-weaning (Table 1). Infants’ ages at each life stage are listed in Table 2. In this study, “introduction of solid foods” indicates that infants had begun to eat solid foods but that nursing still made up the majority of the diet. “Regular consumption of solid foods” indicates that infants consumed solids on a daily basis, in addition to nursing. The transition to “weaning” is marked by a dramatic decrease in the number and duration of nursing bouts allowed by the mother, during which infants consume mostly solid foods but still nurse occasionally. No invasive procedures were performed on the lemurs, and no lemurs were sacrificed as a result of the study. Furthermore, human handlers played no role in assisting the animals during delivery, and only handled infants during exams at the time of sample collection, to minimize human impact on lemur GIT colonization.

**Table 1:** Number of subjects and fecal samples from each lemur species.

Host species	*V*. *variegata*	*L*. *catta*	*P*. *coquereli*	Total
Number of dams	1	3	2	6
Number of infants	3	4	2	9
**Number of infants per dam**	**3**	**1,1,2**	**1,1**	
**Total subjects**	**4**	**7**	**4**	**15**
Parturition	1	3	2	6
Birth	3	3	1	7
Nursing	3	4	2	9
Introduction of solid foods	3	4	2	9
Regular consumption of solid foods	3	4	2	9
Weaning	3	4	2	9
Weaned	3	4	2	9
**Total samples**	**19**	**26**	**13**	**58**

**Table 2:** Age of subjects at each life stage.

Age at life stage	*V*. *variegata*	*L*. *catta*	*P*. *coquereli*
Birth	0–1 days	0–1 days	0–1 days
Nursing	11 days	2 weeks	1–2 weeks
Introduction of solid foods	4 weeks	8 weeks	16 weeks
Regular consumption of solid foods	18 weeks	19 weeks	28 weeks
Weaning	22 weeks	29 weeks	38 weeks
Weaned	36 weeks	43 weeks	54 weeks
Parturition	17 years	11, 6, and 4 years	13 years (both dams)

### Bacterial DNA extraction and sequencing

All fecal specimens were stored in individually labeled Whirl-pak bags and immediately frozen at -80°C to prevent DNA degradation and continued microbial reproduction within feces [29]. The exterior of each frozen sample was cut away; DNA extractions were performed using only fecal matter that had not been exposed to the environment. Microbial DNA was extracted from individual samples using the QIAamp Stool Mini Kit (QIAGEN, Hilden, Germany) following manufacturer guidelines. Bacterial DNA was subsequently visualized on a gel and quantified using a Nanodrop-1000. Two standardized DNA aliquots from each extraction were sent to Argonne National Laboratory for downstream amplification and sequencing. The PCR primers 515F (GTG-CCA-GCM-GCC-GCG-GTA-A) and 806R (GGA-CTA-CHV-GGG-TWT-CTA-AT) were used to amplify the v4 region of 16S rDNA with length ~300bp for 150 pb paired-end sequencing on the Illumina MiSeq platform according to the methods of Caporaso et al. [30]. Technical replicates were sequenced from each DNA sample listed in Table 1 to ensure precision between sequencing efforts. All 116 libraries were barcoded and pooled on a single Illumina MiSeq run. 16S DNA was sequenced in duplicate from 58 fecal samples to create 116 libraries.

### 16S sequence Analyses

Illumina sequencing produced 15,308,697 total reads before filtering. We used ea-utils [31] to join forward and reverse reads, yielding a total of 9,040,165 joined reads. A fastq file containing these joined reads was deposited in the NCBI Sequence Read Archive (Project ID PRJNA270617). Joined 16S reads were analyzed using Quantitative Insights Into Microbial Ecology (QIIME v1.7.0) to classify microbial constituents and compare membership between samples [32,33].

**Fig 2:**
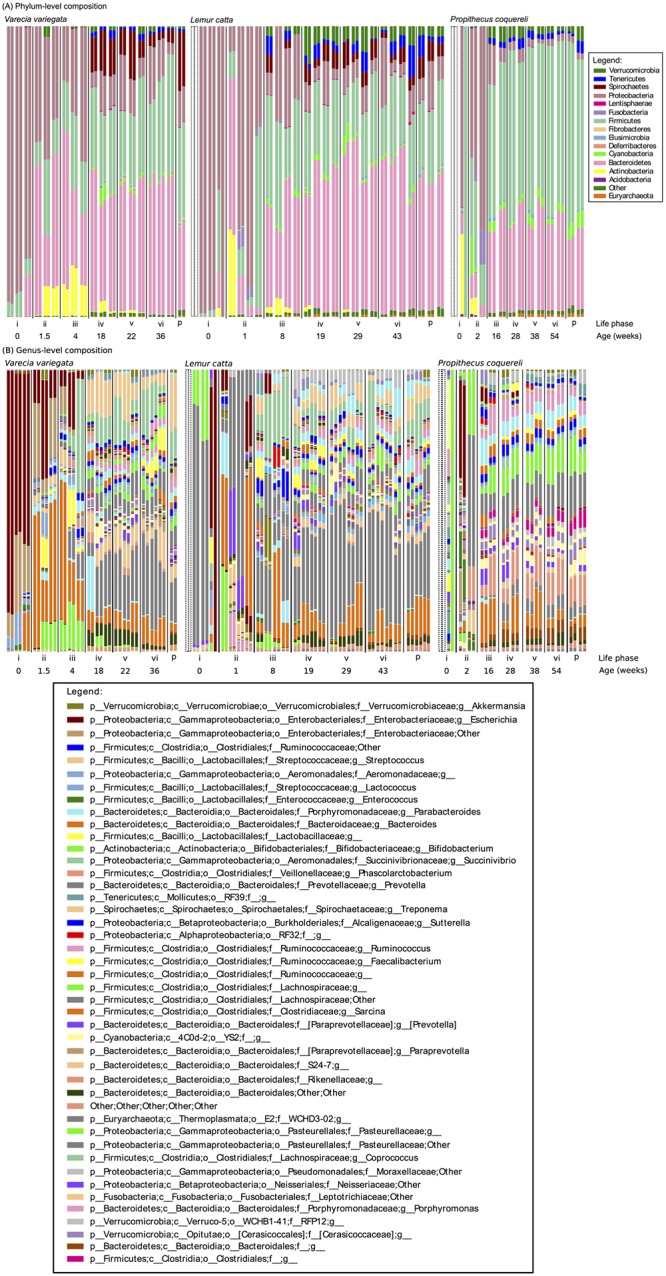
Composition of 16S libraries sequenced in duplicate from 58 fecal samples, collected from 15 lemurs belonging to *Varecia variegata*, *Lemur catta*, and *Propithecus coquereli*. Samples were collected from infants at (i) birth, (ii) nursing, (iii) introduction of solid foods, (iv) regular consumption of solid foods, (v) weaning, (vi) weaned, and from dams at parturition (p). Infants’ ages are listed below each life stage. The samples collected from each species during the introduction of solid foods are also bracketed and indicated with asterisks. Technical duplicates sequenced from each sample are paired and arranged so that infants’ results are repeated in the same order within each life stage and so that dams’ order corresponds to their infants’. Results are shown at the (A) phylum level and (B) genus level.

**Fig 3:**
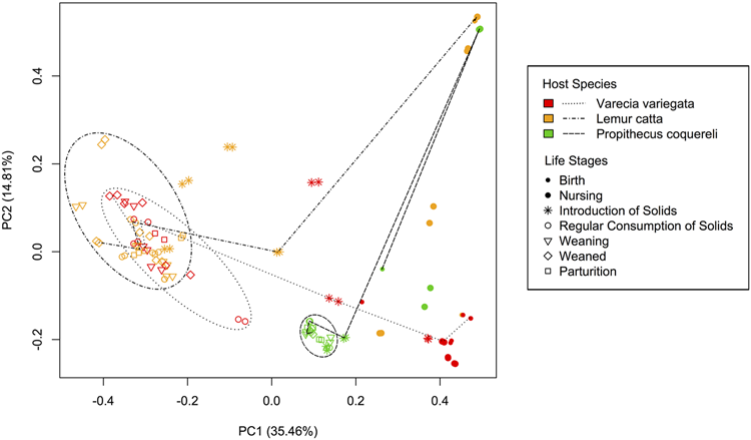
Weighted PCoA of OTU beta diversity between libraries. The distance in PCoA vector space represents community membership (i.e. taxa in Fig 2). Each data point in represents a library (i.e. bar in Fig 2). The distance between points represents unique branches on a phylogenetic tree (i.e. evolutionary history not shared between libraries in Fig 2). Closer points share more branch length, while points more distant from one another have more unique or disparate GMs. Dashed lines connect samples from a representative individual from each species, and represent species-specific trajectories. Representative individuals were chosen based on the completion of longitudinal sampling and quality of sample sequencing. Dashed circles indicate samples collected from animals after they begin consuming solid foods regularly.

We performed quality filtering using default settings and demultiplexed reads using 12 bp barcodes. A total of 5,844,416 sequences were retained after quality filtering in QIIME, giving us an average coverage of 50,297 sequences (standard deviation 15,670) per library. Coverage ranged from 1 sequence (in a library sequenced from a sample collected from *P*. *coquereli* at birth, discussed below) to 82,473 sequences, with a median of 53,668 sequences per library. Duplicate 16S libraries were generally more similar to each other than to libraries sequenced from other samples (Figs 2 and 3), suggesting that the coverage (>50,000 paired-end reads per library after quality filtering in QIIME) adequately captured the taxonomic diversity recovered in each DNA extraction.

Operational taxonomic units (OTUs, a proxy for taxa based on 97% sequence similarity) were picked using UCLUST [34]. Next we calculated the number and frequency of OTUs within each sample and the net difference in OTUs between any two communities to report alpha and beta diversity, respectively. Beta diversity was quantified using weighted UniFrac [35], which calculates the proportion of OTUs unique to each sample. QIIME performs Principle Coordinate Analyses (PCoA) on the weighted GM beta diversity to detect underlying relationships between the microbiota, feeding ecology, host species, and relatedness among hosts.

### Statistical analyses

We used JMP Pro (Version 11, SAS Institute Inc., Cary, NC, USA) to perform a mixed model nested two factor ANOVA, using the model

y = A+B+A*B+∝

where y is the biodiversity index, A is host species (fixed), B is life stage (fixed), A*B is the interaction between host species and life stage, and α is individual nested within species (random).

We used linear contrasts of the Shannon-Weaver and Simpson’s diversity to test for effects of life stage and other host factors. The Shannon-Weaver index calculates biodiversity by taking the log of relative abundance values. This method only slightly reduces the weight of rare species, which may contribute many of the changes between progressive life stages. Simpson’s diversity index, however, squares relative abundance values. Therefore, the weight of rare species is reduced relatively more than the weight of abundant species. This makes Simpson’s diversity more robust in situations where there are many rare species whose changes may potentially disturb overall patterns of change.

We used the adonis{vegan} function in R [36] to calculate the strength and significance of host species and life stage in determining variation in a distance matrix of the weighted UniFrac measurements of beta diversity between all libraries. Adonis is analogous to PERMANOVA [37], a nonparametric method that determines significance through permutations (default = 999) and returns a pseudo F- and p-value. By partitioning distance matrices, PERMANOVA statistically compares the differences in overall community composition between classes of 16S libraries.

Finally, we used Linear discriminant analysis Effect Size (LEfSe, with default settings) to detect bacterial lineages whose frequencies differ significantly between host species at life stages of specific interest, such as the introduction and regular consumption of solid foods. LEfSe is available on the Huttenhower lab Galaxy instance (http://huttenhower.sph.harvard.edu/galaxy), and takes a tab-delimited table of OTU frequencies with class, subclass, and subject headings as input. LEfSe detects differentially distributed lineages with the Kruskall-Wallis test (default alpha value = 0.05), and then checks the consistency of subclass distinctions with the pairwise Wilcoxon text (default alpha value = 0.05). The final linear discriminant analysis (LDA) ranks all differentiating lineages by their effect size (default threshold on logarithmic LDA score = 2.0).

## Results

**Fig 4:**
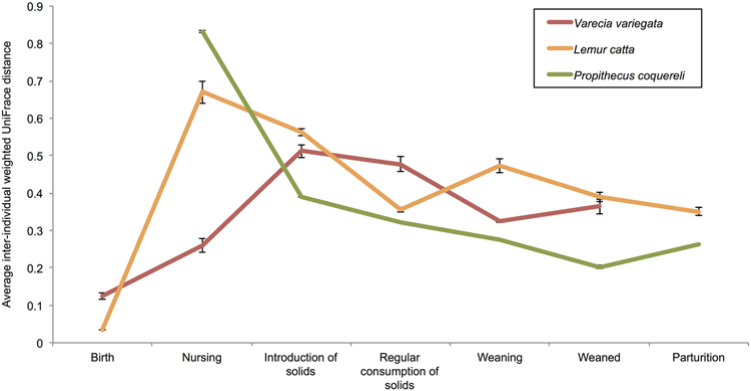
Inter-individual GM variation within species. Weighted UniFrac distances were averaged to plot GM variation at each life stage. The distances between replicate libraries (sequenced from the same fecal sample) were omitted from average calculations. Distance could not be calculated for *Propithecus coquereli* at birth or for *Varecia variegata* at parturition because only one sample was collected at these time points.

**Fig 5:**
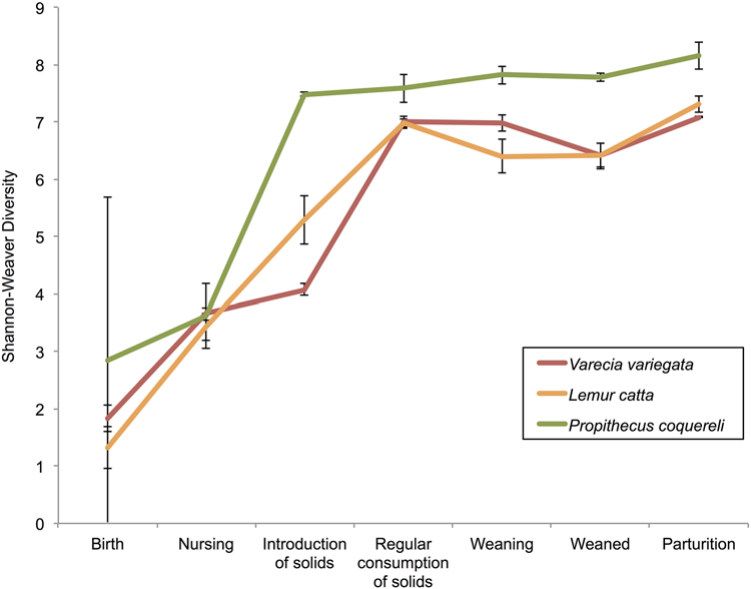
Shannon-Weaver biodiversity values, averaged at each life stage within species.

**Fig 6:**
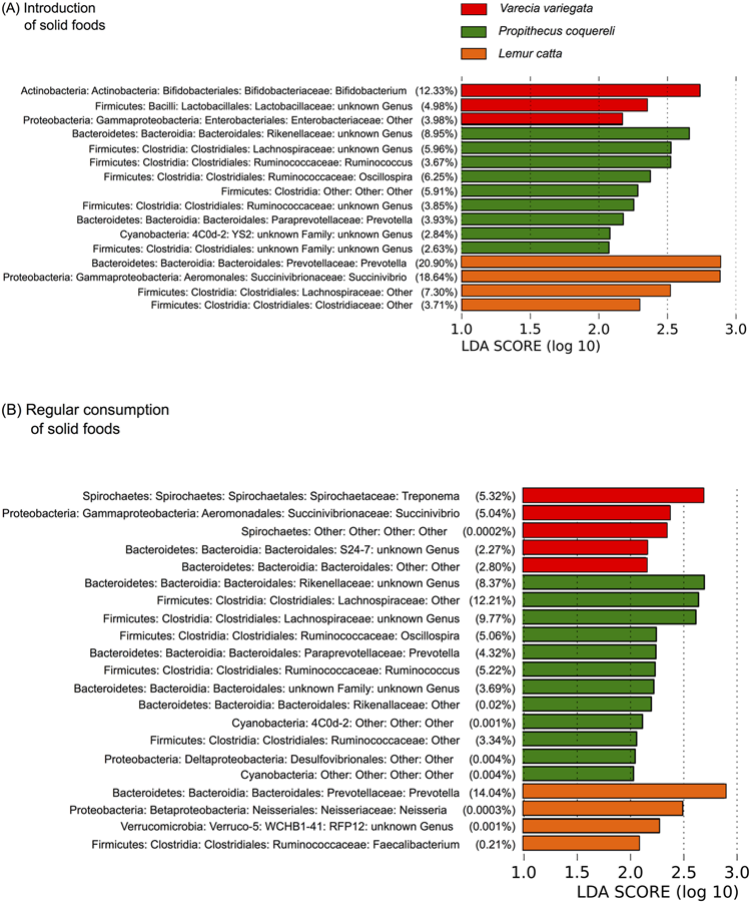
Bacterial lineages that distinguish 16S libraries sequenced from fecal samples collected from lemurs at (A) introduction of solid foods and (B) regular consumption of solid foods. Differentially distributed lineages are ranked based on their linear discriminant analysis effect size. The average percent contribution of each lineage is listed in parentheses.

All statistical tests support the life stage hypothesis. Both alpha diversity measures yielded similar significance; here we present the results of the Shannon-Weaver analyses (Tables 3 and 4). As expected, individuals within species exhibit more variation and less similarity during early life stages (Figs 2 and 3, Table 3). The average weighted UniFrac distance between individuals within species at each life stage also decreases as animals mature (Fig 4). Birth samples contained the lowest bacterial diversity compared to other life stages, with significant increases early in life and continued, though less significant, increases to parturition (Fig 5). Community composition also differed significantly among life stages (Table 4). Birth and nursing samples comprised different bacterial ecological pioneer membership within each lemur species; however, opportunistic colonizers (i.e. Enterobacteria) and lactose digesting bacteria were consistently detected across host species (Fig 2). As lemurs progressed from the introduction to the regular consumption of solid foods, the number of differential lineages detected within each species increased (Fig 6). Yet many of the lineages come from the same clades; and several were retained across life stages, suggesting that they may be members of the native GIT community.

**Table 3:** Statistical tests to determine roles of life stage in shaping GMs.

Shannon-Weaver Contrast	Test	p_*Vv*_	p_*Lc*_	p_*Pc*_	Is hypothesis supported?
$\mu_{i}=\frac{1}{5}\;\left(\mu_{ii}\;+\;\mu_{iii}\;+\;\mu_{iv}\;+\;\mu_{v}\;+\;\mu_{vi}\right)$	Compare birth to other life stages in each species	<0.0001	<0.0001	<0.0001	Yes: alpha diversity is significantly lower in samples from birth than from other life stages in all host species.
μ_i_ = μ_ii_	birth vs. nursing	<0.0001	<0.0001	0.2448	Supported for *V*. *variegata* and *L*. *catta*
μ_ii_ = μ_iii_	Nursing vs, introduction of solids	0.3298	<0.0001	<0.0001	Supported for *L*. *catta* and *P*. *coquereli*
μ_iii_ = μ_iv_	Introduction vs. regular solids	<0.0001	<0.0001	0.8512	Yes
μ_iv_ = μ_v_	Regular solids vs. weaning	0.9551	0.1253	0.6545	Yes: GM composition has not changed significantly.
μ_v_ = μ_vi_	Weaning vs. weaned	0.1971	0.9797	0.9484	Yes: GM composition has not changed significantly.
μ_vi_ = μ_p_	Weaned vs. parturition	0.2810	0.0278	0.4771	Yes: GM composition has not changed significantly.

Diversity should be lowest at birth and increase with age until weaning, plateauing as lemurs approach adulthood and GMs approach climax community makeup.

**Table 4:** Tests to detect GM differences between lemur clades.

Shannon-Weaver Contrast	Test	p-value	Result
$\frac{1}{2}\;\left(\mu_{\left(iv-p\right)}^{Vv}\;+\;\mu_{\left(iv-p\right)}^{Lc}\right)\;=\;\mu_{\left(iv-p\right)}^{Pc}$	Lemuridae versus Indriidae for life stages when solid food is consumed regularly.	p = 0.0002	Average for *P*. *coquereli* (7.837) is significantly higher than Lemuridae (6.822).
$\mu_{\left(iv-p\right)}^{Vv}\;=\;\mu_{\left(iv-p\right)}^{Lc}$	Test for difference between *V*. *variegata* and *L*. *catta* for life stages when solid food is consumed regularly	p = 0.6744	*V*. *variegata* (6.873) does not differ from *L*. *catta* (6.771).
**adonis (life stage)**	Calculate variation due to life stage	p = 0.007	Yes: community composition is significantly different between life stages.

Interestingly, all infant GMs follow the same overall movement and convergence through vector space in Fig 3. For each species, infant GM profiles converge toward the climax community represented here by mothers’ parturition samples within each species as the animals mature (Fig 3). Life stages during which lemurs consume solid food on a daily basis cluster closely in PCoA vector space (Fig 3) and share the same major bacterial constituents (Fig 2), suggesting putative climax communities for each host species (see further discussion below).

*P*. *coquereli* microbial diversity is significantly higher than that of the two species within the Lemuridae (*V*. *variegata*, *L*. *catta)* after solid food is consumed daily (p = 0.0002; Table 3), while the latter two species do not differ significantly from each other (p = 0.6744; Table 4). Furthermore, *P*. *coquereli* hosted a greater number of differential lineages during the introduction (Fig 6) and regular consumption (Fig 6) of solid foods than either lemurid species. Interestingly, the membership within *P*. *coquereli’s* highly diverse GMs was highly conserved. Inter-individual variation, as measured by the average weighted UniFrac distance, was lower in *P*. *coquereli* than either *V*. *variegata* or *L*. *catta* (Fig 4).

## Discussion

Our results indicate that captive diet impacts GM succession (i.e. differences between life stages, Table 3), while species identity of the host accounts for major distinctions among samples across life stages (Table 4). Although *V*. *variegata* and *L*. *catta* are more closely related to each other than either is to *P*. *coquereli*, they are nonetheless separated by a significant period of evolutionary history (approximately 30mya [28]). To contextualize this phylogenetic diversity, there is as much evolutionary distance separating *Varecia* from *Lemur* as there is distance separating humans from marmosets. This long evolutionary separation is manifested by many behavioral, ecological, and phenotypic characteristics. We assume that the gut microbiomes associated with these two species are less constrained evolutionarily than the lemurs’ differently-evolved gut morphologies and other distinguishing physiological and morphological characteristics. We therefore maintain that there has been sufficient opportunity and time for the gut microbiomes of these two species to change independently, so that when they are brought together in captivity and fed similar diets, observed GM similarities are likely to reflect a dietary effect. Diet and phylogeny, however, are clearly confounded. To more thoroughly disentangle the effects of phylogeny and diet, additional species within the lemuriform radiation should be surveyed. A host species’ characteristics are the consequence of the evolutionary impacts of adaptive strategies, such as feeding ecology and life history, and as such, are the ultimate drivers of species’ divergence. While we tested each factor separately, we also discuss interactive effects below.

### Life stage dictates dietary intake, and both drive microbial succession

GM constraint begins at nursing due to dietary restriction: milk is the sole source of food during this stage, unless the infant practices coprophagy (i.e. as practiced by *P*. *coquereli*). The introduction of solid food increases bacterial diversity (Figs 2 and 5) and GM differentiation between host species (Figs 3, 4, and 6) by supplying more diverse nutrients than are available in milk alone. Weaning, in turn, stabilizes dietary diversity (Fig 5) by eliminating milk from the diet and refining the nutrient profile available to GM members, thus decreasing variability within host species (Figs 3 and 4). Regular consumption of solid foods, and weaning especially, seems to drive GM convergence within host species (indicated by high p-values in Table 3, shared bacterial constituents in Fig 2, and decreased average UniFrac distances in Fig 4). All GM libraries exhibit increasing diversity and stability—that is, decreasing variability between individuals and among progressive life stages—with the introduction of solid foods, increasing age, and weaning (Table 3; Figs 2, 3, 4). For example, *V*. *variegata* and *L*. *catta* libraries all contained *Prevotella* after the introduction of solid food, while *P*. *coquereli* libraries from the same life stages all contained four genera within the family Ruminococcaceae (Fig 2).

### Dietary fiber affects microbial diversity

We predicted that *P*. *coquereli* GMs fed a high-fiber captive diet would be significantly different from GMs in *V*. *variegata* and *L*. *catta* fed captive diets high in starches and other soluble carbohydrates. *P*. *coquereli* libraries were the most diverse (Table 4, Fig 5) and contained the most differential bacterial lineages detected by linear discriminant analysis (Fig 6). Of particular interest are four Ruminococcaceae genera detected in *P*. *coquereli* samples after solid food was introduced (Fig 2). Ruminococcaceae were first isolated from ruminant (i.e. cow) gut samples, and have also been detected in folivorous gorillas [13,38]. Thus, the family is likely present in *P*. *coquereli* due to convergent dietary challenges. *P*. *coquereli* consumes the highest fiber diet of the species studied here. Dietary fiber is fermented to produce myriad metabolic byproducts [39], which in turn support the high diversity of gut bacteria detected in our study.

### Species-specific traits distinguish GMs despite diet

While *P*. *coquereli* hosted the GMs with highest diversity, *L*. *catta* and *V*. *variegata* GMs exhibit higher inter-individual variation (Fig 4). LEfSe detected several bacterial lineages that distinguished *V*. *variegata* from *L*. *catta* despite the similar dietary regimes imposed by captivity (Fig 6). Furthermore, putative core microbiomes become apparent for all three species as lemurs approach weaning and adulthood (Fig 3). *L*. *catta* GIT morphology is of intermediate complexity (Fig 1), reflecting adaptation to a higher number of different food items foraged *in situ* compared to *V*. *variegata*. Our results indicate that captive diet plays an integral role in shaping bacterial diversity (Tables 3 and 4, Figs 4 and 5), but also that species-specific traits such as GIT morphology impact the beta diversity observed between the two Lemuridae species (Figs 3 and 6). These findings are consistent with previous work by Ochman et al., which demonstrated that phylogeny is the main driver of gut microbial communities in primates [14].

### Natural diet is tied to host developmental patterns

Primates have fundamentally different life histories as compared to other mammals [40]. Under the protein-richness hypothesis, animals with increased protein intake such as folivores should develop more rapidly compared to frugivores and wean at an earlier age, unless the age at weaning is delayed for optimal coordination with environmental conditions [19,41]. Lemurs exemplify this exception to the rule. Lemurs have evolved estrus cycles comprising fleeting time-windows that occur at species-specific intervals to accommodate their different developmental rates. As a consequence, lemuriform infants wean in synchrony, regardless of species, prior to the arrival of cyclones to Madagascar in March [42]. This island-wide adaptive response to environmental challenges permits development over species-specific intervals, instead of at differing rates. Those intervals are mediated, in part, by food availability and dietary adaptations [43].

*Propithecus* species are highly folivorous [44], consuming natural diets that are enriched for protein and fiber compared to the fruits eaten by *V*. *variegata in situ* [45]. *Propithecus* infants are also more precocious at weaning than frugivorous lemurs [41]; but instead of maturing more quickly, *P*. *coquereli* development takes place over 30 months, compared to 14 months for *V*. *variegata* (DLC records; [42]). These developmental differences can affect many phenotypic traits. For example, folivorous lemurs exhibit adult dentition at weaning to aid in chewing fibrous forages, while frugivorous lemur teeth are less fully developed at weaning [41]. Precocious folivorous lemurs’ delayed weaning may also promote enhanced microbial succession, just as it enables the advanced development of other adaptive traits. Because GMs also aid in fiber digestion, it stands to reason that the patterns of GM development would also vary between lemur species utilizing different feeding strategies.

### Life stage impacts microbial succession differently in each lemur species

The 16S profiles from samples collected after the introduction of solid foods show varying levels of similarity to nursing samples within each lemur species (Figs 2 and 3; Table 3). Specifically, *V*. *variegata* appears to undergo a less profound shift in GM composition with the introduction of solid foods (p = 0.3298; Table 3) compared to the other species in our study (p<0.0001; Table 3). This suggests that early gut colonization has more and longer lasting impact on the GMs in *V*. *variegata*, while GM succession in *L*. *catta* and *P*. *coquereli* progresses further toward the climax communities observed in dams at parturition (Figs 2 and 3). These effects may stem from the species-specific developmental rates discussed above. Alternatively, consumption of solid foods may have less impact on the GM in *V*. *variegata* than in *L*. *catta* or *P*. *coquereli*. A possible explanation might be that the nutritional composition of milk may be more similar to that of the captive diet consumed by *V*. *variegata*. Another possibility is that because *V*. *variegata* has the simplest GIT morphology, it undergoes the least gut tissue development and thus has less transformative impact on GM composition. Importantly, while infants within species exhibit similar colonization trajectories (Fig 3), the 16S signature and infant age at each life stage is distinct between species (Fig 2, Table 2).

GMs from *P*. *coquereli* differ significantly from *V*. *variegata* and *L*. *catta* (p = 0.0002; Table 4), while the sister groups do not differ (p = 0.6744; Table 4). The taxonomic diversity of GMs within each lemur species also appears constrained; that is, many bacterial families are detected in each lemur species that belong to a few orders and are specific to their primate host (Figs 2 and 6), suggesting specialized bacterial radiation appropriate to each host species’ characteristics (i.e. immune system or GIT morphology).

*P*. *coquereli* infants, and indeed the offspring of many herbivores (i.e. horses and rabbits [1,39]), practice maternal coprophagy—they consume their mothers' feces early in life, presumably to inoculate their GIT with beneficial communities. This behavior may explain the only observation during our study wherein the DNA extracted from the sample collected from a *P*. *coquereli* infant at birth yielded two duplicate libraries with inconsistent bacterial membership (Fig 2). One of the libraries contained only one OTU; but the other technical replicate contained higher diversity than other samples collected at birth or even within the nursing sample later collected from the same *P*. *coquereli* infant. This anomalous result may reflect either sequencing error or maternal coprophagy during or shortly after birth. All other duplicates yielded similar 16S results within library pairs.

Sampling from animals with detailed medical records allowed us to verify that hosts were healthy, and therefore ensured that GMs were not affected by illness. Lemur GM profiles appear to converge in accordance with macro-organismal ecology succession trends (Figs 4 and 5), in which the climax community achieves a more conserved composition within species over time. The refined community includes specific members, which are best adapted to the environment and interact with each other within that environment, and thus have greater stability over time.

### Synthesis

All lemur samples from birth and nursing contain predominantly Proteobacteria (Fig 2), similar to GMs in human infants delivered vaginally [7,21]. We find the phyla Firmicutes and Bacteroidetes to dominate all lemur libraries in this study after solid food is consumed (Fig 2), comparable with findings in humans and other primates (Table 5). Lachnospiraceae (Clostridiales: Clostridia: Firmicutes), a major family detected in healthy human gut microbiota [46], was also detected in *P*. *coquereli* birth samples. Interestingly, the gut microbiota sequenced from other non-human primates differ in the distributions of their dominant phyla (Table 5). Specifically, lemurs appear to harbor ratios of Proteobacteria and Firmicutes more similar to *Pan* species than to either *Gorilla* species [14] or to *Nycticebus pygmaeus* (pygmy loris, the only other prosimian whose gut microbiota has been studied to date [17]), while Bacteroidetes shows the opposite relationship.

**Table 5:** Composition of Gut Microbial Communities Characterized in Different Non-human Primates.

	% of phylogenetic lineage^a^									
Bacterial phylum	*Varecia variegata* ^b^	*Lemur catta* ^b^	*Propithecus coquereli* ^b^	*Nycticebus pygmaeus* ^c^	*Gorilla beringei* ^d^	*Gorilla gorilla* ^d^	*Pan paniscus* ^d^	*Pan troglodytes troglodytes* ^d^	*Pan troglodytes schweinfurthii* ^d^	*Pan troglodytes ellioti* ^d^
Actinobacteria	0.54	0.15	—	10.98	1.98	2.94	6.97	3.99	4.34	0.77
Bacteroidetes	40.08	48.52	31.05	41.19	44.98	34.96	19.15	27.86	28.49	19.16
Cyanobacteria	1.18	1.45	3.09	0.28	—	—	—	—	—	—
Elusimicrobia	0.34	0.36	0.36	—	—	—	—	—	—	—
Euryarchaeota	0.27	0.46	0.91	—	—	0.01	0.11	0.01	0.06	0.03
Firmicutes	29.94	24.13	55.39	9.44	4.96	9.93	70.89	53.75	61.66	27.01
Fusobacteria	—	—	—	0.3	—	—	—	0.12	—	—
Lentisphaerae	0.15	0.28	0.07	—	—	—	0.06	0.12	0.02	—
Proteobacteria	11.72	9.08	2.59	30.43	47.98	51.84	1.04	12.59	2.93	52.38
Spirochaetes	11.76	5.18	—	0.50	0.02	0.16	0.40	0.75	1.87	0.07
Tenericutes	1.27	3.55	2.17	—	0.03	0.11	0.37	0.33	0.19	0.46
Verrucomicrobia	1.00	5.07	2.92	1.33	0.03	0.04	0.63	0.32	0.39	0.11
Other	1.26	1.67	1.43	1.12	.03	.02	0.38	0.16	0.07	0.02

^a^Values are proportions of the phylogenetic lineages reported for each next generation sequencing library.

^b^Lemur values are averaged over the following life stages: regular consumption of solids, weaning, weaned, and parturition.

^c^Xu et al. 2013[17] data reported are for metagenomic libraries sequenced from pooled samples from two wild Pygmy Lorises.

^d^Ochman et al. 2010[14] data are values for 16S Sanger sequencing libraries averaged within species for 2 *Gorilla beringei*, 2 *Gorilla gorilla*, 5 *Pan paniscus*, 8 *Pan troglodytes troglodytes*, 5 *Pan troglodytes schweinfurthii*, and 2 *Pan troglodytes ellioti*.

Lemur GMs contained two bacterial lineages associated with humans consuming a distinctly non-Western diet. All lemurs’ GMs contained *Prevotella* after solid food was introduced (Figs 2 and 6), similar to humans from outside the U.S. [2,15,22,23,47]. *V*. *variegata* GMs were also enriched in *Treponema* (Spirochaetes; Figs 2 and 6). GMs isolated from children living in rural Burkina Faso contained both *Prevotella* and *Treponema*, while neither genus was present in European children [47]. The presence of *Prevotella* and *Treponema* indicates bacterial fermentation of plant-derived compounds such as xylene, xylose, and carboxymethylcellulose [48]. Presumably, these bacteria were detected in humans in developing countries due to the higher prevalence of plants and produce in non-Western diets, thus yielding the similarity to those found in lemurs. Accordingly, our results indicate the potential to use lemurs as a model of GM assembly for comparison to human and other primates.

## Supporting Information

S1 Fig Gastrointestinal tract from a red ruffed lemur (*Varecia variegata rubra*). Scale equals 1 cm. Figure is originally published in Campbell et al. 2000 [49], and reused here with permission from John Wiley and Sons.
(TIF)

S2 Fig Gastrointestinal tract from a ring-tailed lemur (*Lemur catta*). Scale equals 1 cm. Figure is originally published in Campbell et al. 2000 [49], and reused here with permission from John Wiley and Sons.
(TIF)

S3 Fig Gastrointestinal tract from a Coquerel’s sifaka (*Propithecus verreaxi coquereli*). Scale equals 1 cm. Figure is originally published in Campbell et al. 2000 [49], and reused here with permission from John Wiley and Sons.
(TIF)

S1 Text ARRIVE checklist. (DOCX)
