# Supplementary figures and images for: Patterns of Gut Bacterial Colonization in Three Primate Species
Source: PLoS One. 2015 May 13;10(5):e0124618. doi: 10.1371/journal.pone.0124618 (PMC4430486; doi:10.1371/journal.pone.0124618)

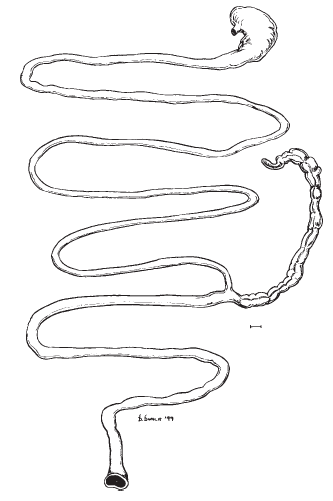

Supplement: S1 Fig — Scale equals 1 cm. Figure is originally published in Campbell et al. 2000 [49], and reused here with permission from John Wiley and Sons. (TIF) [file pone.0124618.s001.tif]

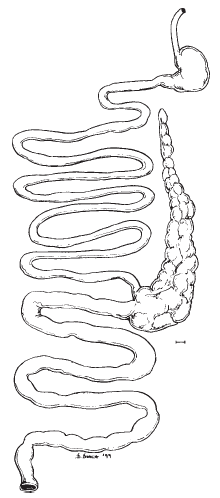

Supplement: S2 Fig — Scale equals 1 cm. Figure is originally published in Campbell et al. 2000 [49], and reused here with permission from John Wiley and Sons. (TIF) [file pone.0124618.s002.tif]

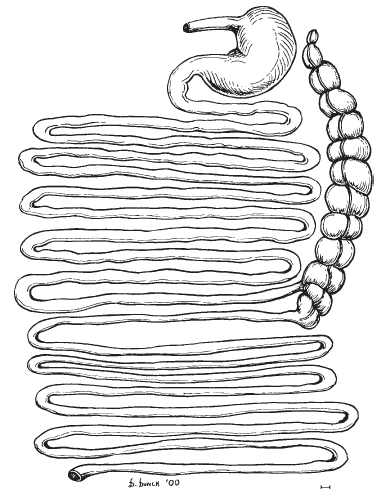

Supplement: S3 Fig — Scale equals 1 cm. Figure is originally published in Campbell et al. 2000 [49], and reused here with permission from John Wiley and Sons. (TIF) [file pone.0124618.s003.tif]
