# Supplementary material for: Patterns of Gut Bacterial Colonization in Three Primate Species
Source: PLoS One. 2015 May 13;10(5):e0124618. doi: 10.1371/journal.pone.0124618 (PMC4430486; doi:10.1371/journal.pone.0124618)
Supplement: S1 Text — (DOCX) [file pone.0124618.s004.docx]

**The ARRIVE Checklist**

**TITLE**

**1 Provide as accurate and concise a description of the content of the article as possible.**

Patterns of gut bacterial colonization in three primate species

**ABSTRACT**

**2 Provide an accurate summary of the background, research objectives (including details of the species or strain of animal used), key methods, principal findings, and conclusions of the study.**

Host fitness is impacted by trillions of bacteria in the gastrointestinal tract that digest fiber, defend against pathogenic invasion, and facilitate development of the intestines and other organs, including the brain. Gut microbiota are inextricably tied to host development and, by extension, all components of life history. During development, microbial colonization primes the gut metabolism and physiology, thereby setting the stage for adult nutrition and health. The ecological rules governing microbial succession are poorly understood, however. In this study, we examined the relationship between host lineage, diet, and life stage and gut microbiota characteristics in three primate species (infraorder, Lemuriformes). Fecal samples were collected from captive lemur mothers and their infants, from birth to weaning. Microbial DNA was extracted and the v4 region of 16S rDNA was sequenced on the Illumina platform using protocols from the Earth Microbiome Project. Here, we show that colonization proceeds along different successional trajectories in developing infants from species with differing dietary and ecological profiles: frugivorous *Varecia variegata*, generalist *Lemur catta*, and folivorous *Propithecus coquereli*. Our analyses reveal community membership and succession patterns consistent with previous studies of human infants, suggesting that lemurs may serve as a useful model of microbial ecology in the primate gut. Each lemur species exhibits distinct species-specific bacterial diversity signatures correlating to life stages and life history traits, implying that gut microbial community assembly primes developing infants at species-specific rates for their respective adult feeding strategies

**INTRODUCTION**

**Background**

**3 a. Include sufficient scientific background (including relevant references to previous work) to understand the motivation and context for the study, and explain the experimental approach and rationale.**

**b. Explain how and why the animal species and model being used can address the scientific objectives and, where appropriate, the study’s relevance to human biology.**

Research on the human gut microbiome (GM) has revealed that the GM has profound impacts on the human phenotype, ranging from cognition to locomotion. Furthermore, the GM is associated with health and disease states throughout life, and there is specific interest in discerning when and how GM manipulation might be most beneficial to the host. Yet it remains difficult to tease apart the relative significance of host characteristics in shaping the human GM, and to establish the general evolutionary and ecological “rules” by which the GM is developed and maintained. Previous research has either focused on a single species (i.e. human, mouse, or rat) over several time points, or compared many species or individuals at a single time point. We chose to study the composition and community development of GMs from birth to weaning across three lemur species: *Varecia variegata*, *Lemur catta*, and *Propithecus coquereli*. The primate clade Lemuriformes is sister to the haplorrhine primates (monkeys, apes and humans). Lemurs have evolved diverse GIT morphologies to adapt to species-specific feeding ecologies, making them an ideal group for teasing apart the potentially divergent effects of phylogenetic history and diet on GM community structure and for examining the associated community signatures for potential effects on host health and nutritional uptake.

**Objectives**

**4 Clearly describe the primary and any secondary objectives of the study, or specific hypotheses being tested.**

- We analyzed bacterial 16S rDNA reads amplified from positively identified fecal samples to resolve GM membership at the genus level and to compare succession patterns between individuals and across species.
- By controlling certain environmental variables such as dietary intake and recording relevant long-term metadata, we investigate the contributions of host life stage, diet, and GIT morphology to the host-gut microbial relationship and the process of community assembly.
- We use summary statistics of taxonomic richness, membership, and diversity to test the following hypotheses.
- **H­_1_: If the succession process in lemurs is similar to that described in humans, then microbial diversity should be lowest in samples from birth and increase with age until weaning, with decreasing intraspecific variability as individuals approach adulthood and their GMs approach the climax community. We refer to this as the “life stage” hypothesis.**
- **H­_2_: If host diet determines gut community structure, then GMs from *V. variegata* and *L. catta* should be more similar to each other than to *P. coquereli*. We refer to this as the “diet-driven” hypothesis.**
- **H_3_: If GIT morphology has a significant impact on microbial composition, then GMs from *V. variegata* and *L. catta* should differ despite the similarity of their diets. We refer to this as the “morphology-driven” hypothesis.**

**METHODS**

**Ethical statement**

**5 Indicate the nature of the ethical review permissions, relevant licenses (e.g. Animal [Scientific Procedures] Act 1986), and national or institutional guidelines for the care and use of animals, that cover the research.**

All animal procedures were reviewed and approved by the Duke University IACUC under protocol number A282-11-11.

**Study design**

**6 For each experiment, give brief details of the study design, including:**

**a. The number of experimental and control groups.**

**b. Any steps taken to minimise the effects of subjective bias when allocating animals to treatment (e.g., randomisation procedure) and when assessing results (e.g., if done, describe who was blinded and when).**

**c. The experimental unit (e.g. a single animal, group, or cage of animals).**

**A time-line diagram or flow chart can be useful to illustrate how complex study designs were carried out.**

1. Samples were collected from mother-infant groups belonging to three species: *Varecia variegata* (3 triplets born to 1 dam), *Lemur catta* (2 singletons and 2 twins born to 3 dams), and *Propithecus coquereli* (2 singletons born to 2 dams).
2. Standardized DNA samples were sent to Argonne National Labs for “blinded” sequencing of technical replicates.
3. Each fecal sample was sequenced in duplicate. Samples were collected from positively identified individuals at known developmental lifestages. The table below lists the number of samples collected within each host species and at each life stage.

| **Host species** | ***V. variegata*** | ***L. catta*** | ***P. coquereli*** | **Total** |
| --- | --- | --- | --- | --- |
| Number of dams | 1 | 3 | 2 | 6 |
| Number of infants | 3 | 4 | 2 | 9 |
| **Total subjects** | **4** | **7** | **4** | **15** |
| Parturition | 1 | 3 | 2 | 6 |
| Birth | 3 | 3 | 1 | 7 |
| Nursing | 3 | 4 | 2 | 9 |
| Introduction of  solid foods | 3 | 4 | 2 | 9 |
| Regular consumption of solid foods | 3 | 4 | 2 | 9 |
| Weaning | 3 | 4 | 2 | 9 |
| Weaned | 3 | 4 | 2 | 9 |
| **Total samples** | **19** | **26** | **13** | **58** |

**Experimental procedures**

**7 For each experiment and each experimental group, including controls, provide precise details of all procedures carried out. For example:**

**a. How (e.g., drug formulation and dose, site and route of administration, anaesthesia and analgesia used [including monitoring], surgical procedure, method of euthanasia). Provide details of any specialist equipment used, including supplier(s).**

**b. When (e.g., time of day).**

**c. Where (e.g., home cage, laboratory, water maze).**

**d. Why (e.g., rationale for choice of specific anaesthetic, route of administration, drug dose used).**

1. Fresh fecal samples were collected from subjects either during routine handling or from enclosures immediately after excretion. Samples were collected from infants with a sterile cotton swab during handling in the few cases when defecation did not occur.
2. All samples were collected between 7:30am and 4:30pm, generally according to each species’ specific gut passage rates and/or circannual rhythms. (i.e. *P. coquereli* defecate voluntarily between 7:30-9am; *L. catta* between 10:30-noon; and *V. variegata* between 2-2:30pm.)
3. Samples were collected either in Duke Lemur Center veterinary examination rooms (during routine handling of infants during early-life weigh-in and check-up) or from the home cage.
4. Fecal samples provide a non-invasive sample of the conglomerate gut microbiome. No additional handling was required to collect the samples. For both reasons, this research sampling method is ideal for the study of endangered primates.

**Experimental animals**

**8 a. Provide details of the animals used, including species, strain, sex, developmental stage (e.g., mean or median age plus age range), and weight (e.g., mean or median weight plus weight range).**

**b. Provide further relevant information such as the source of animals, international strain nomenclature, genetic modification status (e.g. knock-out or transgenic), genotype, health/immune status, drug- or test naıve, previous procedures, etc.**

*V. variegata* infants: 1 female, 2 males

*L. catta* infants: 2 females, 2 males

*P. coquereli* infants: 1 female, 1 male

| **Age at life stage** | ***V. variegata*** | ***L. catta*** | ***P. coquereli*** |
| --- | --- | --- | --- |
| Birth | 0-1 days | 0-1 days | 0-1 days |
| Nursing | 11 days | 2 weeks | 1-2 weeks |
| Introduction of  solid foods | 4 weeks | 8 weeks | 16 weeks |
| Regular consumption of solid foods | 18 weeks | 19 weeks | 28 weeks |
| Weaning | 22 weeks | 29 weeks | 38 weeks |
| Weaned | 36 weeks | 43 weeks | 54 weeks |
| Parturition | 17 years | 11, 6, and 4 years | 13 years (both dams) |

**Housing and husbandry**

**9 Provide details of:**

**a. Housing (e.g., type of facility, e.g., specific pathogen free (SPF); type of cage or housing; bedding material; number of cage companions; tank shape and material etc. for fish).**

**b. Husbandry conditions (e.g., breeding programme, light/dark cycle, temperature, quality of water etc. for fish, type of food, access to food and water, environmental enrichment).**

**c. Welfare-related assessments and interventions that were carried out before, during, or after the experiment.**

1. Housing- indoor enclosures with access to adjoining outdoor runs; number of cage companions for our subjects varies from 2-10, depending on the dam’s mothering experience and social dynamics within the group. The typical indoor cage is 10'h X 7.5'w X 7.0'l. The typical outdoor enclosure is 10'h X 7.5'w X 14'l. The number of indoor and outdoor cages a group has depends upon the size of the group. Usually, it's spaced so that each group member could have his/her own indoor and outdoor area if needed (i.e., for a group of 5 animals they'd have 5 connected indoor and 5 connected outdoor enclosures to freely move between).
2. Husbandry conditions- The lighting conditions for our diurnal lemurs is simply a natural North Carolina photoperiod. Since at least half of their space is outdoors and with windows in each indoor enclosure, it gets dark when the sun goes down and light when the sun comes up. There is also fluorescent lighting, which is turned on 7am-5pm (minimum) while staff are present. Temperature inside 70^o^F ± 2 ^o^F; water quality- Durham city water has no violations and averages pH range of 7.4-7.7; environmental enrichment- nestlet for singly housed and breeding; Food: The following are representative dietary regimes per individual. *V. variegata* receive 80-100g Lab Diet**®** #5038 and 1 cup fruit-veggie mixture. *L. catta* receive 60g Lab Diet**®** #5038and ¾ cup fruit-veggie mixture. *P. coquereli* receive 75g Mazuri**®** Leaf Eater # 5675, 30g greens, 30g veggies, 10g nuts/beans, and 150g leaves. Amounts may vary depending on individual health, weight, and reproductive status. Fresh water provided in clean metal bowls daily and replenished as needed. Enrichment provided in form of bamboo or wooden climbing stuctures, sturdy plastic play houses, firehoses and ropes for climbing, and rotating novel objects (i.e. scents, paper mache objects, puzzles with treats inside, etc.).
3. Not applicable

**Sample size**

**10 a. Specify the total number of animals used in each experiment and the number of animals in each experimental group.**

**b. Explain how the number of animals was decided. Provide details of any sample size calculation used.**

**c. Indicate the number of independent replications of each experiment, if relevant.**

a.

| **Host species** | ***V. variegata*** | ***L. catta*** | ***P. coquereli*** | **Total** |
| --- | --- | --- | --- | --- |
| Number of dams | 1 | 3 | 2 | 6 |
| Number of infants | 3 | 4 | 2 | 9 |
| **Total subjects** | **4** | **7** | **4** | **15** |
| Parturition | 1 | 3 | 2 | 6 |
| Birth | 3 | 3 | 1 | 7 |
| Nursing | 3 | 4 | 2 | 9 |
| Introduction of  solid foods | 3 | 4 | 2 | 9 |
| Regular consumption of solid foods | 3 | 4 | 2 | 9 |
| Weaning | 3 | 4 | 2 | 9 |
| Weaned | 3 | 4 | 2 | 9 |
| **Total samples** | **19** | **26** | **13** | **58** |

b. All mother-infant groups from the 2011 breeding season (2012 infant cohort) were included in the study. The species were chosen for their diverse ecological and GIT morphological adaptations.

c. Two standardized aliquots of DNA extracted from each of the samples in the table above were sent to Argonne National Labs for sequencing. (Technical replicates)

**Allocating animals to experimental groups**

**11 a. Give full details of how animals were allocated to experimental groups, including randomisation or matching if done.**

**b. Describe the order in which the animals in the different experimental groups were treated and assessed.**

1. Animals were grouped by species and by developmental lifestage.
2. Samples were collected at each developmental life stage.

**Experimental outcomes**

**12 Clearly define the primary and secondary experimental outcomes assessed (e.g., cell death, molecular markers, behavioural changes).**

Primary: identification of gut bacterial taxa, and calculation of microbiome diversity

Secondary: comparison of succession and microbial community composition between individuals, across host species, and correlation with host traits and metadata

**Statistical methods**

**13 a. Provide details of the statistical methods used for each analysis.**

**b. Specify the unit of analysis for each dataset (e.g. single animal, group of animals, single neuron).**

**c. Describe any methods used to assess whether the data met the assumptions of the statistical approach.**

Sequencing data was analyzed using Quantitative Insights Into Microbial Ecology (QIIME v1.7.0). Operational taxonomic units (OTUs, a proxy for taxa based on 97% sequence similarity) were picked using UCLUST. Alpha diversity was calculated using Simpson’s diversity and Shannon-Weaver diversity indices. Beta diversity was quantified using weighted UniFrac. We used JMP**®** Pro (Version 11, SAS Institute Inc., Cary, NC, USA) to perform a mixed model nested two factor ANOVA, using the model

 , where y is the biodiversity index, A is host species (fixed), B is life stage (fixed), A*B is the interaction between host species and life stage, and α is individual nested within species (random). We used the adonis{vegan} function in R (analogous to PERMANOVA) to calculate the strength and significance of host species and life stage in determining variation in a distance matrix of the weighted UniFrac measurements of beta diversity between all libraries. Finally, we used Linear discriminant analysis Effect Size (LEfSe) to detect bacterial lineages whose frequencies differ significantly between host species at life stages of specific interest, such as the introduction and regular consumption of solid foods. LEfSe detects differentially distributed lineages with the Kruskall-Wallis test, then checks the consistency of subclass distinctions with the pairwise Wilcoxon text. The final linear discriminant analysis ranks all differentiating lineages by their effect size.

**RESULTS**

**Baseline data**

**14 For each experimental group, report relevant characteristics and health status of animals (e.g., weight, microbiological status, and drug- or test-naıve) before treatment or testing (this information can often be tabulated).**

All animals analyzed were in good health.

**Numbers analysed**

**15 a. Report the number of animals in each group included in each analysis. Report absolute numbers (e.g. 10/20, not 50%).**

**b. If any animals or data were not included in the analysis, explain why.**

Please refer to #6. Two infants were not sampled at birth to minimize stress during a critical phase of new dam – infant bonding.

**Outcomes and estimation**

**16 Report the results for each analysis carried out, with a measure of precision (e.g., standard error or confidence interval).**

| **Lifestage** | **Species** | **Shannon-Weaver** | **Std Error** | **Simpson** | **Std Error** |
| --- | --- | --- | --- | --- | --- |
| Birth | V variegata | 1.8334033 | 0.30245765 | 0.48326694 | 0.377671 |
| Nursing | V variegata | 3.6571396 | 0.30245765 | 0.79863668 | 0.377671 |
| Intro solids | V variegata | 4.0748426 | 0.30245765 | 0.84263162 | 0.377671 |
| Reg solids | V variegata | 7.0042163 | 0.30245765 | 0.96182572 | 0.377671 |
| Weaning | V variegata | 6.9801481 | 0.30245765 | 0.96037693 | 0.377671 |
| Weaned | V variegata | 6.4260502 | 0.30245765 | 0.93279419 | 0.377671 |
| Parturition | V variegata | 7.0828037 | 0.52387201 | 0.06643556 | 0.06541453 |
| Birth | L catta | 1.320878 | 0.30674185 | 0.37494924 | 0.0377671 |
| Nursing | L catta | 3.4299384 | 0.261936 | 0.76275318 | 0.3270727 |
| Intro solids | L catta | 5.2891154 | 0.261936 | 0.91454174 | 0.3270727 |
| Reg solids | L catta | 6.9708543 | 0.261936 | 0.97067304 | 0.3270727 |
| Weaning | L catta | 6.3995234 | 0.261936 | 0.92889088 | 0.3270727 |
| Weaned | L catta | 6.4089335 | 0.261936 | 0.94881559 | 0.3270727 |
| Parturition | L catta | 7.3064246 | 0.30245765 | 0.97460551 | 0.377671 |
| Birth | P coquereli | 2.857549 | 0.5386107 | 0.47402596 | 0.06541453 |
| Nursing | P coquereli | 3.6208805 | 0.37043345 | 0.77711591 | 0.04625506 |
| Intro solids | P coquereli | 7.4863057 | 0.37043345 | 0.98045317 | 0.04625506 |
| Reg solids | P coquereli | 7.5845613 | 0.37043345 | 0.98137988 | 0.04625506 |
| Weaning | P coquereli | 7.8190102 | 0.37043345 | 0.98282887 | 0.04625506 |
| Weaned | P coquereli | 7.7851065 | 0.37043345 | 0.98645394 | 0.04625506 |
| Parturition | P coquereli | 8.1593743 | 0.37043345 | 0.98807341 | 0.04625506 |

**Adverse events**

**17 a. Give details of all important adverse events in each experimental group.**

**b. Describe any modifications to the experimental protocols made to reduce adverse events.**

There were no adverse events.

**DISCUSSION**

**Interpretation/scientific implications**

**18 a. Interpret the results, taking into account the study objectives and hypotheses, current theory, and other relevant studies in the literature.**

**b. Comment on the study limitations including any potential sources of bias, any limitations of the animal model, and the imprecision associated with the results.**

**c. Describe any implications of your experimental methods or findings for the replacement, refinement, or reduction (the 3Rs) of the use of animals in research.**

We found increasing microbial diversity but decreasing interindividual variation as lemur infants developed from birth to weaning. Our results suggest that diet (and especially dietary fiber) drives the increase of microbial diversity (in concurrence with previous studies). Furthermore, species-specific GIT morphology may be responsible for species-specific differences between microbiomes in lemurs fed similar diets. We used captive lemurs, and thus our findings may not be entirely relevant to free-ranging wild populations. Our sampling was also limited to the number of dams bred in 2011 and infants born in the resulting 2012 cohort.

**Generalisability/translation**

**19 Comment on whether, and how, the findings of this study are likely to translate to other species or systems, including any relevance to human biology.**

This initial characterization of the lemur microbiome identified several bacterial constituents shared with other (human and non-human) primates. Furthermore, through statistical comparison of gut community composition, we were able to identify which bacterial lineages are differentially represented in each host lemur species. Finally, this study lays the groundwork for comparison between the healthy gut microbiome and lemurs challenged with intestinal pathogens, which would presumably disrupt the native community with implications for health and captive management.

**Funding**

**20 List all funding sources (including grant number) and the role of the funder(s) in the study.**

This research was supported in part by Duke Biology departmental Grant-in-Aid and the Duke Lemur Center Director’s Fund. The funders had no role in study design, data collection and analysis, decision to publish, or preparation of the manuscript.
